# Fermented Gold Kiwi Improves Gastrointestinal Motility and Functional Constipation: An Animal Study and Human Randomized Clinical Test

**DOI:** 10.3390/nu16213778

**Published:** 2024-11-03

**Authors:** Jihye Choi, Hwal Choi, Yuseong Jang, Hyeon-Gi Paik, Hyuck-Se Kwon, Seon Mi Shin, Jeung Seung Lee, Bumseok Kim, Jungkee Kwon

**Affiliations:** 1 Biosafety Research Institute, College of Veterinary Medicine, Jeonbuk National University, 79 Gobong-ro, Iksan-si 54596, Republic of Korea; jyyye@naver.com (J.C.); narcism077@naver.com (H.C.); bskims@jbnu.ac.kr (B.K.); 2 Department of Laboratory Animal Medicine, College of Veterinary Medicine, Jeonbuk National University, 79 Gobong-ro, Iksan-si 54596, Republic of Korea; rhdejr17@naver.com (Y.J.); gusrl007@naver.com (H.-G.P.); 3 R&D Team, Food and Supplement Health Claims, Vitech, Wanju 55365, Republic of Korea; sek2gun@nate.com; 4 Department of Internal Medicine, College of Korean Medicine, Semyung University, Semyong-ro 65, Jecheon-si 27136, Republic of Korea; bunggujy21@hanmail.net; 5 Daesang Jongga R&D Product Division, 178 Magokjungang-ro, Gangseo-gu, Seoul 07789, Republic of Korea; js0614@daesang.com

**Keywords:** constipation, loperamide, fermented gold kiwi, probiotics, neurotransmitter

## Abstract

Constipation is a functional disorder of the gastrointestinal system characterized by difficult bowel movements, infrequent defecation, reduced water content, and hard stools. This study aims to evaluate the preventive effects of fermented gold kiwis (FGK) on loperamide-induced constipation in rats and investigate its efficacy in improving constipation symptoms in human patients through a randomized clinical trial. In the animal study, FGK was administered orally at doses of 50, 125, and 250 mg/kg to constipated rats for two weeks, resulting in significant improvements in constipation parameters. FGK increased serum serotonin and acetylcholine levels and suppressed increases in serum dopamine concentration. FGK also upregulated mRNA expression of the serotonin-synthesizing receptors 5-HT3R and 5-HT4R and suppressed the expression of the dopamine 2-receptor (D2R) in the duodenum. Furthermore, FGK inhibited inflammatory cytokines such as tumor necrosis factor (TNF)-α, interleukin (IL)-1β, and IL-6. In the clinical trials, the improvement in constipation symptoms was evaluated using the gastrointestinal symptom rating scale (GSRS). Clinical trial participants reported significant improvements in constipation symptoms after receiving FGK. These findings suggest that FGK effectively relieves constipation in both animals and humans, indicating its potential as an effective dietary supplement.

## 1. Introduction

Constipation is a functional disorder of the gastrointestinal system characterized by difficult bowel movements, infrequent defection, reduced water content, and hard stools [1,2]. It affects between 3% and 15% of the general population and is associated with various diseases [3]. In Korea, the prevalence of constipation is 12.5%. The number of individuals affected by constipation increased from approximately 580,000 in 2011 to 640,000 in 2020 [4]. A meta-analysis of global studies found that the prevalence of chronic constipation ranged from 14% to 30%, reflecting differences in diagnostic criteria and population characteristics [5]. The annual medical costs for a patient suffering from chronic constipation are estimated to be USD 11,911 in the US, highlighting its significant economic burden and the negative impact on individuals’ quality of life [6,7]. The quality of life for patients suffering from constipation is significantly low [8,9]. Most patients do not achieve satisfactory treatment outcomes with the administration of laxatives, creating a need for therapeutic alternatives.

The pathophysiology of constipation, as it is currently understood, is unclear. However, gastrointestinal (GI) motility is generally considered to be multifactorial, involving inflammation and secretory dysfunction, which, in turn, have significant influences on the enteric nervous system (ENS) [10,11]. GI motility is regulated by the interstitial cells of Cajal and the ENS, both of which affect constipation and bowel motility [10,12]. The neurotransmitter 5-hydroxytryptamine (5-HT) is crucial for regulating various physiological functions related to bowel movement, such as GI motility, smooth muscle contraction, and neural signaling [13,14]. Drugs used to treat constipation include serotonin 5-HT4 receptor agonists, which are effective but can cause nausea and vomiting [2,15]. In addition, while linaclotide is effective in treating constipation, it can lead to diarrhea [16]. Therefore, there is a need for more effective and safer treatments for patients with functional constipation and GI motility issues.

Kiwi is a nutritionally rich fruit containing not only vitamin C, vitamin E, and dietary fiber but also several antioxidants and bioactive compounds [17]. Kiwis provide additional physiologically active substances, such as organic acids and polyphenols, which have antioxidant and anti-cancer properties and influence intestinal health [18]. Gold kiwi is generally relatively rich in calcium, beta-carotene, lutein, and other nutrients compared with the better-known green kiwi [19]. In hospitals and elderly care facilities in New Zealand, it is a common practice to administer kiwi juice (Kiwi Crush) to present and alleviate constipation. In addition, Kiwi Crush is also known among patients at Wellington Hospital to be an effective treatment for constipation [20]. Several studies, including our previous research [21,22], have found that gold kiwis have protective effects on the gastric mucosa and therapeutic effects on gastrointestinal disorders such as peptic ulcers and duodenal ulcers. In patients with irritable bowel syndrome (IBS), kiwi consumption is associated with an increase in stool frequency and volume, as well as improved bowel function [23]. In addition, kiwi consumption can reportedly alleviate symptoms in patients with constipation-predominant IBS and help maintain gastrointestinal function by regulating gut microbiota and the inflammatory process [24]. A recent clinical trial study found that the daily consumption of two gold kiwis can be effective in treating constipation in patients [25].

While kiwis have a high nutritional value, processing activities such as peeling increase ethylene production and respiration rates, which promote microbial spoilage and result in a short shelf life [26]. However, fermentation processes, such as those used in wine and vinegar, have been shown to result in higher nutrient levels compared with those associated with the processing of juice [27]. Kiwis fermented with *Lactobacillus plantarum* reportedly contain higher levels of antioxidant compounds and exhibit greater antioxidant activity [28]. Similarly, in a previous study, we found that fermented gold kiwis (FGK) also showed increased antioxidant potential, such as the increased activity of 2,2-diphenyl-1-picrylhydrazyl and 2,2′-azino-bis(3-ethylbenzothiazoline-6-sulfonic acid) [21]. The primary objective of this study was to investigate the effects of FGK (Kiwibiotics) on gastric emptying, gastrointestinal motility, and the induction of constipation in a rat model, using five strains of lactic acid bacteria. In addition, among the dyspeptic patients recruited for the study, we observed and evaluated any changes in those who exhibited symptoms of constipation after receiving a daily dose. These findings are expected to provide new insights and add value regarding the potential of FGK to promote gastrointestinal motility and alleviate constipation.

## 2. Materials and Methods

### 2.1. Preparation of the Fermented Gold Kiwi Sample

The commercial FGK sample used in this study, Kiwibiotics, was provided by Vitech (Wanju, Republic of Korea). Gold kiwi puree was obtained from Namuang Foods (Jeju, Republic of Korea). Both the fresh and pureed forms of gold kiwis were used as a substrate for fermentation by probiotics. Initially, the kiwis were washed thoroughly with distilled water (DW) to eliminate impurities, then they were finely chopped with their peel included. The chopped kiwis and puree were subjected to fermentation under slating and degreasing conditions. Five strains of bacteria were used for the fermentation process (Table 1).

**Figure 1:**
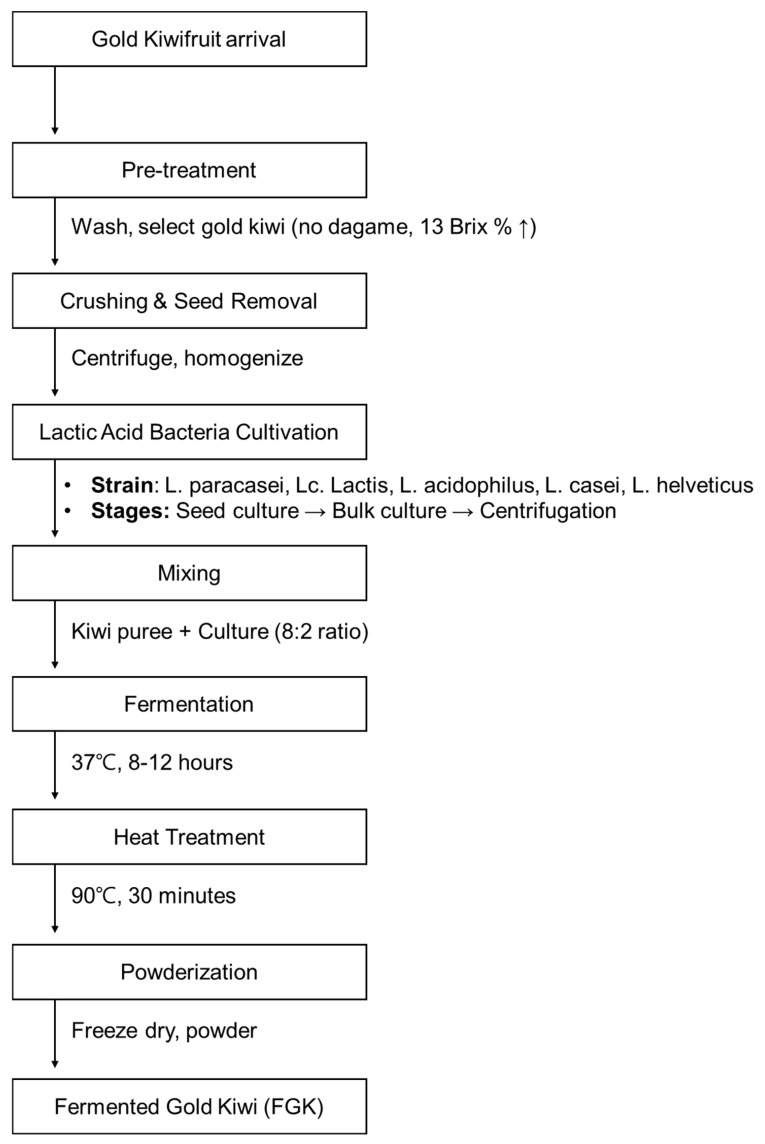
Flowchart of the fermentation process of gold kiwi.

After fermentation, the samples were uniformly mixed, inoculated onto BCP plate-count agar (EIKEN Chemical Co., Ltd., Tokyo, Japan), and incubated at 37 °C for 48 h. These isolated strains were mixed with gold kiwi puree at a ratio of 2:8 and cultured at 37 °C for 8–12 h. After completing the fermentation process, the samples were subjected to heat treatment at 90 °C for 30 min for sterilization purposes. Following concentration and freeze-drying (Figure 1), the resulting powder was used for an animal study and a human clinical trial. FGK is a post-biotic product as it contains no active bacteria or Vitamin C.

The sample was freeze-dried and stored at −70 °C until use. The components of FGK are listed in Table 2.

### 2.2. Animals

Six-week-old male Sprague-Dawley (SD) rats were purchased from Damool Sciences (Daejeon, Republic of Korea) and allowed to acclimate to laboratory conditions for 1 week before being used in the study. During the experimental period, the animals were given free access to food and water. All rats were handled in compliance with the guidelines set by the Jeonbuk National University Institutional Animal Care and Use Committee, which approved the study protocol (approval number: NON2024-060).

#### 2.2.1. Analysis of Anti-Secretory Activity in Pyloric Ligation

After 24 h of fasting, the SD rats were randomly distributed into five groups (n = 6): a vehicle control, a positive control group that received sucralfate at 50 mg/kg, and experimental groups that received 50, 125 and 250 mg/kg of the active agent (labeled FGK 50, FGK 125, and FGK 250). The rats were anesthetized with isoflurane 30 min after sample administration. The abdominal cavity was opened, and pyloric ligation was performed. The rats were moved to a cage after suturing the surgical site. The rats were euthanized with isoflurane 6 h after pyloric ligation, at which time gastric juices were collected from the stomach. A gastric juice supernatant was obtained after 10 min of centrifugation at 2000× *g*. The volume of gastric juice and the pH were measured using a pH meter (Hanna, Woonsocket, RI, USA). Acidity was measured by titration with 0.05 N NaOH, using phenolphthalein as an indicator. Total acidity was calculated according to the following formula [29]:

Total acidity (mEq/6h) = Vol. of gastric juice (mL) × Vol. of NaOH (mL) × normality of 0.05 N NaOH

In addition, a supernatant of the gastric juices was used to measure pepsin activity. Pepsin activity was calculated according to the following formula [30]:

Pepsin activity (Units/mL) = [(A_280_ sample − A_280_ blank) × dilution factor] ÷ t × v

t = assay incubation time in min

v = volume of gastric juice (mL) added

#### 2.2.2. Measurement of Gastric Emptying and the Establishment of an Animal Model

Cisplatin is a drug that is widely used to treat malignant tumors. However, the high-dose administration of cisplatin can cause side effects such as kidney and gastrointestinal damage, making its use in a clinical setting challenging [31]. The administration of cisplatin in animals induces abdominal distension, dyspepsia, and delayed gastric emptying [32]. The rats fasted for 48 h before the experiment. All rats were randomly assigned to 1 of 6 groups (n = 6): normal controls (vehicle + DW), a model control group (vehicle + cisplatin), a positive control group (itopride 30 mg/kg + cisplatin), and experimental groups receiving 50, 125, and 250 mg/kg of FGK + cisplatin. Each substance was prepared with 3% hydroxyl propyl methyl cellulose (HPMC) as a vehicle, and the cisplatin was dissolved in DW. Simultaneously, the normal group received DW, while the other groups received 10 mg/kg of cisplatin intraperitoneally. One hour after the administration of each substance, a liquid meal containing 1.5% HPMC and 0.5% phenol red was administered orally. Finally, the rats were euthanized using isoflurane, and samples were obtained from the rats. The percentage of gastric emptying was calculated using the following formula [33]:

Gastric emptying (%) = 1 − [(concentration of phenol red in the test stomach)/(concentration of phenol red in a standard stomach)] × 100

#### 2.2.3. Measurement of Gastrointestinal Motility and the Establishment of an Animal Model

Atropine blocks the action of the parasympathetic nervous system and has side effects such as reduced intestinal peristalsis [34]. The rats, which fasted for 48 h before the experiments, were randomly assigned into a total of 6 groups (n = 6): normal controls (vehicle + DW), model control group (vehicle + atropine), positive control group (mosapride 10 mg/kg + atropine), and 3 experimental groups (50, 125, and 250 mg/kg of FGK + atropine). Each substance was prepared with 3% HPMC as a vehicle, and the atropine was dissolved in DW at a concentration of 1 mg/kg. All experimental groups except the normal group received atropine intraperitoneally, while the normal group received an equal volume of DW. One hour after administration of the substances, 1 mL of fluorescein isothiocyanate (FITC)-dextran, prepared at 6.25 mg/mL, was administered orally to each rat.

After administration of the agents, the rats were anesthetized and euthanized with isoflurane 15 min later to measure the amount of FITC-dextran remaining in the small intestine. The small intestine was excised and the ends were secured to prevent the contents from spilling out. The excised intestine was divided into 10 segments and the geometric center (GC) of each segment was measured. Each segment was homogenized with 3 mL of 0.05 M Tris buffer, then centrifuged at 800 g for 5 min. The supernatant was collected, and fluorescent signals were measured using a microplate reader (Synergy, 2, BioTek instrument, Winooski, VT, USA) at 490–520 nm. The GC value was calculated using the following formula [35]:

G.C. = ∑ (% of total fluorescent signal per segment × segment number)/100

#### 2.2.4. Induction of Constipation and the Study Design

**Figure 2:**
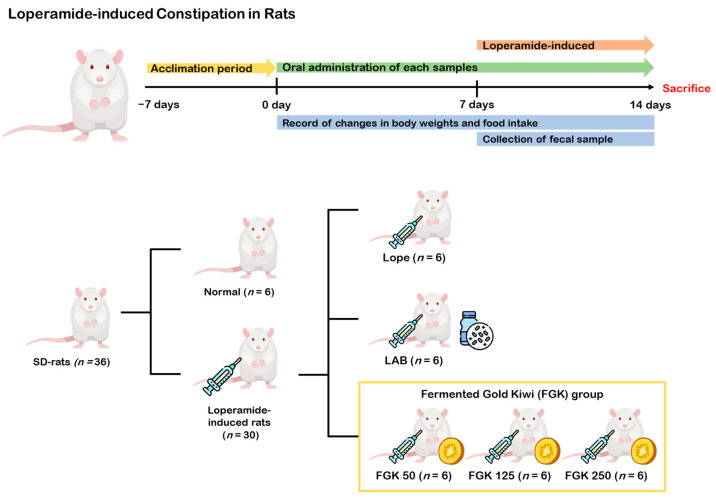
Loperamide-induced constipation in rats: experimental design.

Loperamide, which is used to treat diarrhea, induces delayed colonic transit by inhibiting intestinal water secretion, resulting in decreased stool frequency and increased colonic contractions [3,36]. All rats were randomly assigned to either an experimental group or the normal control group, with 6 rats in each group: normal control (vehicle + DW), model control group (Lope = vehicle + loperamide), positive control group (LAB = lactic acid bacteria-included fermentation of FGK, 22 × 10^8^ CFU/day + loperamide), FGK 50 (FGK 50 mg/kg + loperamide), FGK 125 (FGK 125 mg/kg + loperamide), FGK 250 (FGK 250 mg/kg + loperamide). All groups received their respective samples once a day for 14 days via oral administration. In addition, from experimental days 8 to 14, the rats were given loperamide (20 mg/kg) once daily for 7 days to induce constipation (Figure 2). The normal group received DW. During the experimental period, we recorded body weights and collected fecal samples to measure the moisture content and other parameters. At the end of the experimental period, all rats were anesthetized using isoflurane (Ifran Liq, Hana Pharm Co., Ltd., Seoul, Republic of Korea), and blood samples were collected. The rats were then euthanized using diethyl ether (DAEJUNG Chemicals & Metals, Siheung, Republic of Korea), and stomach, large intestine, and fecal samples were collected from the euthanized rats.

### 2.3. Measurements of Body Weight, Food Intake, Fecal Numbers, and Moisture

We measured the changes in body weight and food intake before and after the administration of loperamide to rats with induced constipation. Body weight and food intake were measured twice a week and fecal numbers were measured once a week before loperamide treatment, after which they were measured daily. The fecal moisture content was calculated by immediately measuring the weight of feces collected over 24 h and drying them in a 60 °C dry oven for 72 h. The weight of the dry feces and fecal moisture was calculated using the following formula:

Fecal moisture content (%) = [(Fecal wet weight − Fecal dry weight)/Fecal wet weight] × 100

### 2.4. Measurement of Serum Neurotransmitter Factors

The rats were anesthetized using isoflurane (Ifran Liq., Hana Pharm Co., Ltd., Seoul, Korea), and their blood was collected from the abdominal vein. The obtained blood was centrifuged at 800× *g* for 15 min, and the resulting serum was used for analysis. Serum serotonin concentration was measured using a rat serotonin ELISA kit (MBS166089, MyBioSource, San Diego, CA, USA). Serum dopamine concentration was determined using a dopamine ELISA kit (BA-E-5300R, ImmunSmol SAS, Pessac, France). Serum acetylcholine was measured using an acetylcholine assay kit (ab65345, Abcam, Cambridge, UK). All assays were conducted according to the manufacturer’s instructions.

### 2.5. Quantitative Real-Time PCR

RNA was isolated from the stomach, colon tissue, and fecal samples of constipated rats using an RNeasy Mini Kit (74104, QIAGEN, Valencia, CA, USA). Isolated RNA was transcribed to cDNA using PrimeScript RT reagent with gDNA Eraser (RR047A, TaKaRa Bio Inc., Otsu, Japan). Additionally, quantitative real-time polymerase chain reaction (qRT-PCR) amplification was conducted using a BioFACT 2X Real-Time PCR Master Mix, including SYBR Green I and high Rox reference dye (DQ385-40h, BIOFACT, Daejeon, Republic of Korea) by a two-step cycler and a StepOne Real-Time PCR System (Applied Biosystems, Middlesex County, MA, USA). All expression levels were quantified by the ∆∆Ct method and normalized to GAPDH expression without bacterial groups. The bacterial groups were normalized to total bacteria. The primer sequences are shown in Table 3.

### 2.6. Human Clinical Study

A randomized, double-blind, placebo-controlled trial was conducted over 8 weeks to evaluate gastrointestinal symptom improvements in dyspeptic adults, with a secondary aim of assessing constipation symptom changes. Random assignment was carried out in a blinded manner for all participants. The protocol for the clinical trial was approved by the Institutional Review Board of Semyung University Korean Medicine Hospital in Jechon, Korea (IRB number: SMJOH-2023-02). This study was registered at the Clinical Research Information Service (CRIS) at the start of the clinical trial study, registered on 27 January 2023 (CRIS number: KCT009414). The trial was approved by the Semyung University Korean Medicine Hospital’s ethical review board, in accordance with local regulations. The study consisted of a 1-week lead-in period (≤7 days) followed by an 8-week (56 ± 5 days) treatment and follow-up period. Eligible participants were randomly assigned to either the FGK group, which consumed Kiwibiotics (FGK), or the Plaeco group, which received a placebo comparator (citrus concentrate). All participants underwent treatment and follow-up over the 8-week period, with a total of 5 scheduled visits: visit 1 (Day ≤ −7, screening), visit 2 (Day 0, randomization, baseline), visit 3 (Day 14 ± 3), visit 4 (Day 28 ± 5), and visit 5 (Day 56 ± 5) [37].

#### 2.6.1. Participants and Recruitment

Participants were recruited through announcements posted on the homepage and bulletin board of Semyung University Korean Medicine Hospital. Eligibility was determined based on the Rome IV criteria, which include: (1) bothersome postprandial fullness, (2) bothersome early satiation, (3) bothersome epigastric pain, (4) bothersome epigastric burning, and (5) no evidence of structural disease likely to explain the symptoms [38]. Some participants (*n* = 105) exhibited constipation symptoms, and these patients were evaluated separately for changes in constipation following FGK intake.

Participants were recruited only if they met all of the following criteria: (1) aged between 19 and 70, (2) reporting at least one symptom of dyspepsia* classified as Rome IV but not requiring prompt medication, (3) reporting at least two major symptoms tracked by the Korean version of the Nepean Dyspepsia Index (NDI-K), with a moderate score (3 points) or higher and a total score of 6 or higher. Additionally, participants were primarily recruited based on the Rome IV criteria for dyspepsia, but some participants also exhibited symptoms of constipation. These individuals were included in this study, and changes in their constipation symptoms were evaluated separately following FGK intake for 8 weeks. Participants were excluded if they had undergone gastrointestinal surgery within the past 6 months, had a relevant medical history, or required continuous medication that could induce gastritis. Those with severe diseases, such as advanced cerebrovascular disease, heart disease, or malignancies, were also excluded. Participants with uncontrolled diabetes or hypertension, creatinine levels more than twice the upper limit of normal levels, or aspartate transaminase and alanine transaminase levels more than 3 times the upper limit of normal levels were excluded. Individuals who had participated in another clinical trial within the previous 3 months, had food allergies, or were pregnant or breastfeeding were also excluded from the study.

#### 2.6.2. Study Protocol

The 100 eligible participants were randomized (1:1 ratio) to receive 1 of the following treatments for 8 weeks: FGK (*n* = 48, excluded *n* = 2) or the placebo (*n* = 45, excluded *n* = 5). Based on the results of previous animal experiments [21], the daily intake of FGK for each participant was determined. In this study, the participants received either FGK or the placebo. The placebo was formulated to match the FGK preparation in appearance, taste, and volume (5 mL packets). The placebo contained a citrus concentrate without any active ingredients, ensuring that the treatment and the placebo were indistinguishable to maintain blinding. Both FGK and the placebo were taken orally once daily for 8 weeks. This was in line with the standard procedures outlined in the CONSORT guidelines for randomized, double-blind, placebo-controlled trials. Participants visited the hospital at visit 2 (baseline) and visit 5 (endpoint) for safety monitoring and to complete a gastrointestinal symptom rating scale (GSRS) questionnaire. To maintain the double-blinded nature of the study, measurements were taken by a third party who was not involved in the study. Adherence was defined as the participant consuming at least 80% of the prescribed treatment.

#### 2.6.3. Clinical Symptoms

The evaluation was conducted based on the GSRS questionnaire, which is considered the most reliable and valid method for assessing GI symptoms [39,40]. The GSRS is a 15-item, disease-specific tool that evaluates gastrointestinal symptoms across five domains: Reflux, Abdominal Pain, Indigestion, Diarrhea, and Constipation. In this study, the focus was primarily on the Constipation domain and on the sensation of incomplete bowel emptying, as these were most relevant to assessing the effects of FGK on lower gastrointestinal symptoms. These focused evaluations helped clarify the impact of FGK on symptom clusters that are specifically associated with constipation-related discomfort and functional outcomes. Although the GSRS consists of 15 items across 5 domains, this study focused specifically on 2 items related to constipation symptoms, which were deemed the most relevant to our research objectives. Each item was assessed using a 7-point Likert scale ranging from “no discomfort at all” to “very severe discomfort”. However, to address the limitations of focusing on individual items, we also performed an analysis of the relevant domain scores, in line with the GSRS guidelines, to provide a more comprehensive evaluation of the participant’s gastrointestinal symptoms.

### 2.7. Statistical Analysis

#### 2.7.1. Animal Study

All results were expressed as the mean ± standard error of the mean (*n* = 6), and a one-way analysis of variance (ANOVA) was conducted using Prism 5 software (GraphPad Software, San Diego, CA, USA), followed by a post hoc analysis using Tukey’s test. Results were considered statistically significant at the *p* < 0.05 level.

#### 2.7.2. Clinical Trial Study

All results were expressed as the mean ± standard deviation, and all statistical analyses were conducted using SAS software (version 9.4., SAS, Wake County, NC, USA). Comparisons of changes in the GSRS symptom scores between the two groups (FGK and Placebo) over time (V1 and V5) were analyzed using a repeated-measures ANOVA, followed by a test of the interactions between the two groups over time.

## 3. Results

### 3.1. Effects of FGK on Gastric Emptying and Gastrointestinal Motility

#### 3.1.1. Gastric Emptying

**Figure 3:**
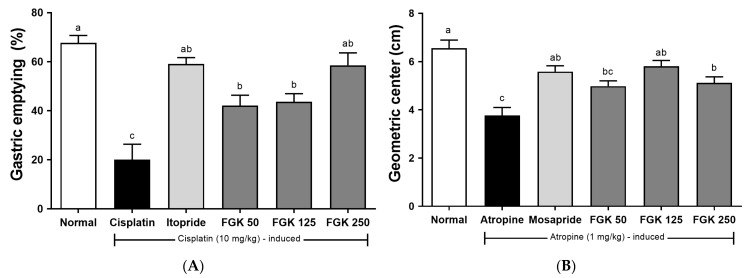

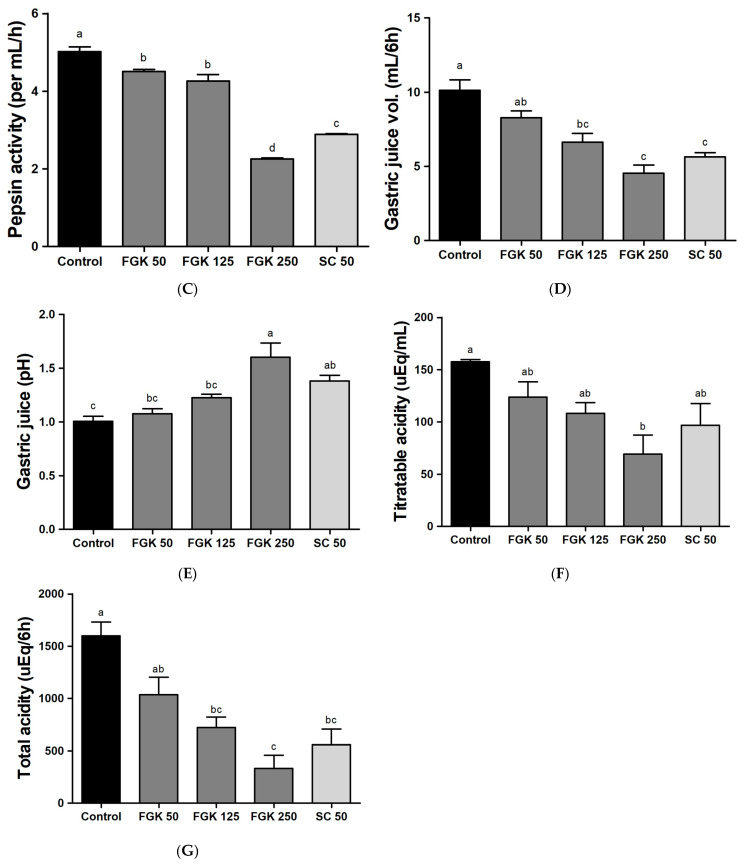
Effects of FGK on gastrointestinal motility in SD rats. (**A**) Gastric emptying in cisplatin-induced SD rats; (**B**) geometric center in atropine-induced SD rats; (**C**) pepsin activity; (**D**) gastric juice volume; (**E**) gastric juice pH; (**F**) titratable acidity; (**G**) total acidity. Data are presented as the mean ± SEM (n = 6). ^a–d^ Different letters indicate significant differences between groups at the *p* < 0.05 levels. Statistical analysis was performed using a one-way ANOVA, followed by Tukey’s post hoc test to determine the specific differences between groups.

The effects of FGK on cisplatin-induced delayed gastric emptying are presented in Figure 3A. Compared with the normal group, gastric emptying in the cisplatin group decreased from 67.7% ± 5.0% to 20.3% ± 11.1%. In the FGK 50, 125, and 250 groups, the percentages were 40.9% ± 6.9%, 43.7% ± 5.9%, and 57.9% ± 7.7%, respectively, while the itopride group (positive control) achieved emptying rates of 59.1% ± 4.5%. Significant recovery was observed in all FGK-treated groups compared with the cisplatin group, while the FGK 250 group showed results comparable to those of the positive control group.

#### 3.1.2. Gastrointestinal Motility

The small intestine was divided into 10 segments, and the amount of FITC-dextran in each segment was measured to calculate the GC, which was used to determine changes in gastrointestinal transit. In Figure 3B, a comparison between the normal group and atropine groups showed a tendency for decreased GC values in the atropine group. Significant results were observed in the experimental FGK 125 and 250 groups, compared with the atropine group. The administration of FGK appeared to induce the recovery of gastrointestinal transit, indicating a need for further experiments on the related mechanisms.

#### 3.1.3. Total Acidity and Pepsin Activity

As shown in Figure 3C, rats treated with FGK experienced significantly decreased pepsin activity levels compared with the control group. In addition, the FGK 250 group exhibited lower gastric juice volume and increased gastric values compared with the control group (Figure 3D,E). Moreover, titratable acidity and total acidity levels were significantly lower in the FGK 250 group compared with the control group (Figure 3F,G). These results indicate that the consumption of FGK can lead to anti-secretion activity in pyloric-ligation rat models.

### 3.2. Effects of Body Weight and Feed Intake in Induced-Constipation Rats

No significant differences in body weight were evident among the groups (Table 4) during the experimental period. However, feed intake decreased in the LOPE group compared to the Normal group, to 19.55 ± 0.02 g/day and 20.05 ± 0.17 g/day, respectively. The LAB group, as well as the FGK 125 and 250 groups, had significantly higher feed intakes compared with the Lope group, indicating a potential positive effect of FGK and LAB on appetite or metabolic function in this model. These results are similar to those in previous studies, indicating that FGK consumption improves feed intake in loperamide-induced constipation rat models.

### 3.3. Effects of FGK on Fecal Parameters in Induced-Constipation Rats

**Figure 4:**
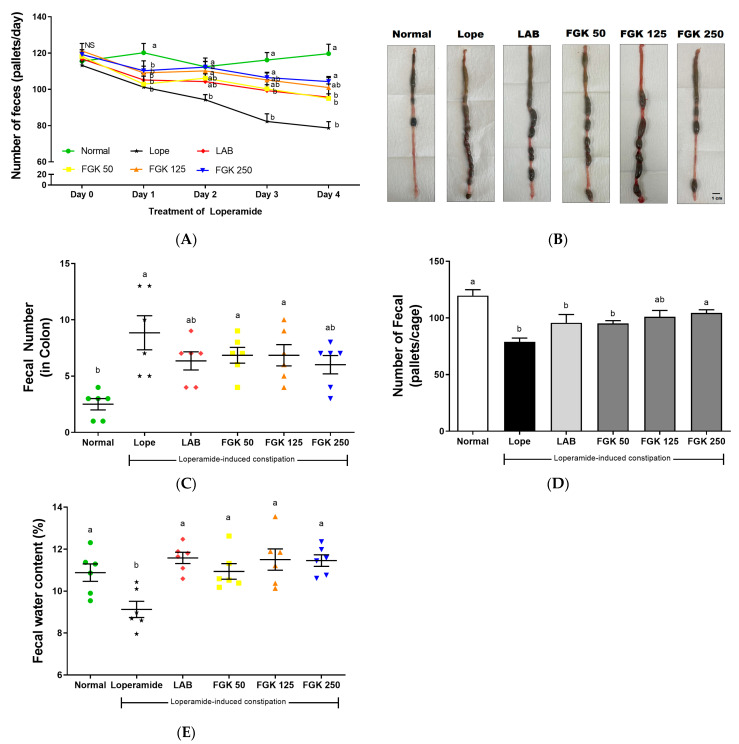
Change in fecal parameters in loperamide-induced constipation rats. (**A**) Number of feces during the experimental period after injecting loperamide; (**B**) representative pictures of the colons of rats in each group; (**C**) number of feces in the colon, as counted, with symbols representing individuals rat fecal counts; (**D**) number of feces in the cages, as counted in the final day; (**E**) fecal water content on the final day, with symbols indicating individual rat measurements. Data are presented as mean ± SEM (n = 6). ^a,b^ Different letters indicate significant differences between groups at the *p* < 0.05 level. Statistical analysis was performed using a one-way ANOVA, followed by Tukey’s post hoc test to determine specific differences between the groups.

Our results indicated the successful establishment of a constipation animal model using loperamide treatment for 5 days. The Lope group exhibited a significant increase in fecal numbers from day 1 to 4 compared with the Normal group (Figure 4A). In comparison to the Lope group, the FGK 250 group exhibited a significant increase in fecal numbers from day 2 to day 4. On the final experimental day, the Lope group showed a substantial accumulation of fecal matter in the colon (Figure 4B). However, the FGK 250 group experienced a dose-dependent decrease in fecal numbers. At the end of the experiment, the Lope group exhibited the largest number of retained feces in the colon, whereas the FGK 250 group had fewer retained feces compared with the Lope group (Figure 4C). The number of feces per cage observed over 24 h (2 rats/cage) was lowest in the Lope group, while FGK administration resulted in a dose-dependent increase (Figure 4D). Additionally, fecal moisture content was significantly higher in the FGK groups compared with the Lope group (Figure 4E).

### 3.4. Effects of FGK on Neurotransmitters in Induced-Constipation Rats

**Figure 5:**
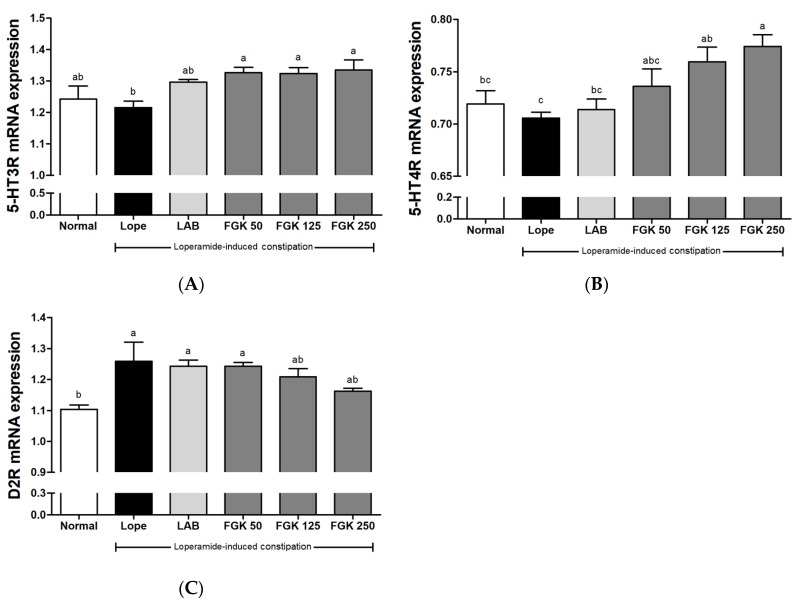
Effects of (**A**) 5-HT3R, (**B**) 5-HT4R, and (**C**) D2R mRNA expression in the duodenum. All data were calculated using the ∆∆Ct method of quantitative RT-PCR, and transcript expression was normalized using the *GAPDH* housekeeping gene. Data are presented as the mean ± SEM (n = 6). ^a–c^ Different letters indicate significant differences between groups at the *p* < 0.05 level. Statistical analysis was performed using a one-way ANOVA, followed by Tukey’s post hoc test to determine specific differences between the groups.

qRT-PCR analysis was performed to evaluate the effects on the receptor of 5-HT3, 5-HT4, and D2R in rat duodenal tissue (Figure 5). In rats with loperamide-induced constipation, the levels of 5-HT3 and 5-HT4 were lower than in the Normal group (Figure 5A,B). In the FGK 125 and 250 groups, the expression of 5-HT3R and 5-HT4R increased compared with the Lope group. In addition, when assessing the effects of the expression of D2R, which is secreted by the gastric mucosa, the Lope group showed a significant increase compared with the Normal group (Figure 5C). Although the increase was not statistically significant, a general decrease in D2R expression was observed in the FGK 250 group (*p* = 0.2473).

**Figure 6:**
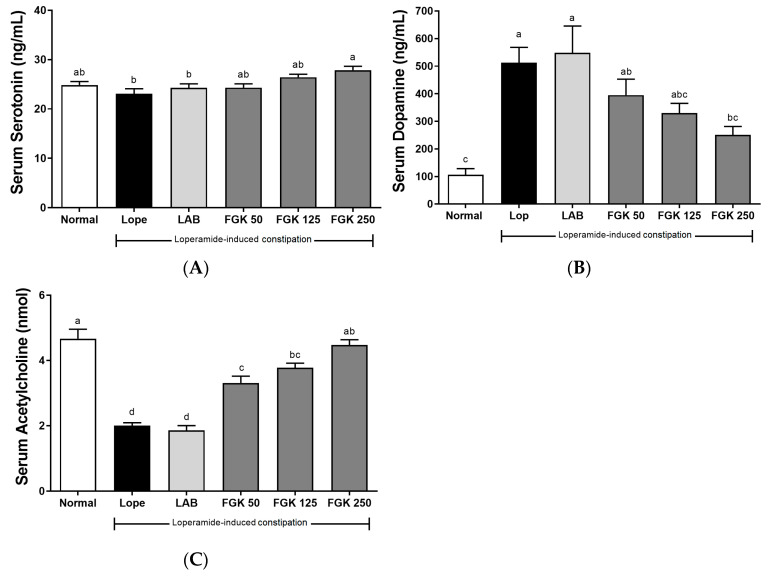
Effects of FGK on the serum neurotransmitter in loperamide-induced constipation rats: (**A**) serotonin; (**B**) dopamine; (**C**) acetylcholine. Data are presented as the mean ± SEM (n = 6). ^a–d^ Different letters indicate significant differences between groups at the *p* < 0.05 level. Statistical analysis was performed using a one-way ANOVA, followed by Tukey’s post hoc test to determine specific differences between the groups.

The levels of 5-HT (serotonin) were lower in the Lope group following loperamide induction, but the reduction was not significant when compared with the Normal group (Figure 6A). However, the FGK 250 group showed increased serum serotonin levels compared with the Lope group. Serum dopamine levels were significantly higher in the Lope group compared with the Normal group (Figure 6B). FGK administration resulted in a dose-dependent decrease, with significantly lower levels observed in the FGK 250 group. Serum acetylcholine levels were significantly lower in the Lope group compared with the Normal group, but a significant increase was seen in the FGK 250 group (Figure 6C).

### 3.5. Effects of FGK on Cytokine Expression in Induced-Constipation Rats

**Figure 7:**
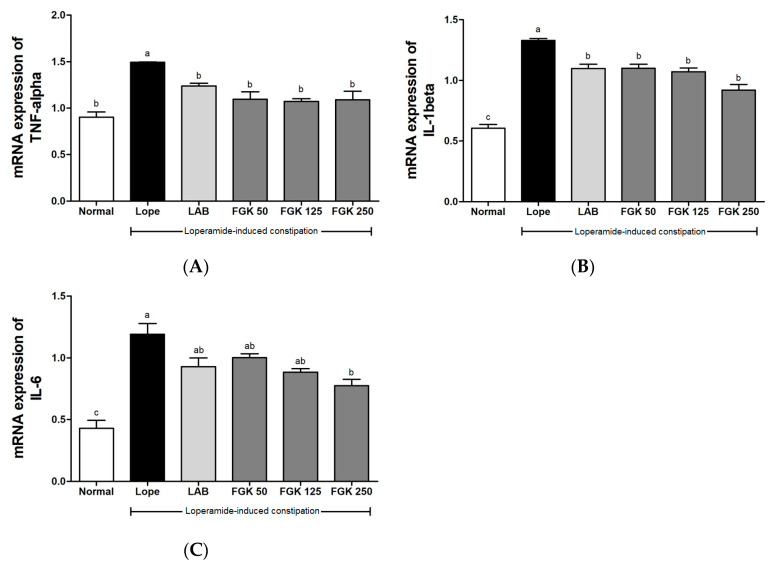
Effects of FGK on mRNA expression of inflammatory cytokines in loperamide-induced constipation rats. (**A**) TNF-α; (**B**) IL-1β; (**C**) IL-6. Data are presented as the mean ± SEM (n = 6). ^a–c^ Different letters indicate significant differences between groups at the *p* < 0.05 level. Statistical analysis was performed using a one-way ANOVA, followed by Tukey’s post hoc test to determine specific differences between the groups.

The concentrations of inflammatory cytokines, such as TNF-α, IL-1β, and IL-6, in the colons of rats with constipation induced by loperamide were measured. A notable impact was evident on the inflammatory cytokines (TNF-α, IL-1β, and IL-6) due to loperamide-induced constipation in the Lope group (Figure 7). The upregulation of cytokine mRNA levels resulting from constipation gradually decreased in a dose-dependent manner following the administration of FGK. The significantly reduced expression of TNF-α, IL-1β, and IL-6 was observed in the FGK 250 group compared with the Lope group. These findings indicate that FGK treatment may alleviate the inflammatory response in the colonic tissue induced by constipation symptoms, showing a protective effect on the compromised colonic barrier.

### 3.6. Effects of FGK on mRNA Expression of Fecal Bacteria Groups in Induced-Constipation Rats

**Figure 8:**
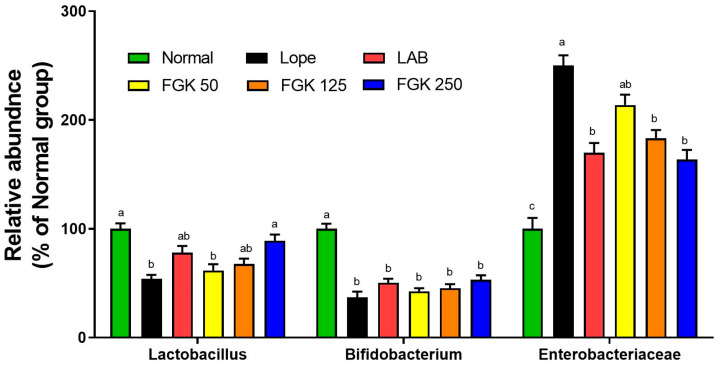
Effects of FGK on the mRNA expression of fecal bacteria groups in loperamide-induced constipation rats. Data are presented as the mean ± SEM (n = 6). ^a–c^ Different letters indicate significant differences between groups at the *p* < 0.05 level. Statistical analysis was performed using a one-way ANOVA, followed by Tukey’s post hoc test to determine specific differences between the groups.

We performed an evaluation of the bacterial communities in feces by analyzing mRNA expression to confirm the equilibrium of the host’s bacterial microbiome. Loperamide-induced constipation resulted in a notable decrease in the abundance of *Lactobacillus* and *Bifidobacterium* in the group treated with Lope compared with the Normal group (Figure 8). In the Lope group (loperamide-induced only group), the fecal *Lactobacillus* levels were decreased compared with the Normal group, while the reduced *Lactobacillus* levels were significantly increased in the FGK 250 group. However, no disparity in the presence of *Bifidobacterium* was evident (*p* = 0.1538). The levels of Enterobacteriaceae were found to have increased significantly in the Lope group compared with the Normal group (*p* < 0.01), whereas there was a notable decrease in the levels of *Enterobacteriaceae* in the FGK 125 and 250 groups (*p* < 0.05).

### 3.7. Human Clinical Study Demographic Characteristics

**Figure 9:**
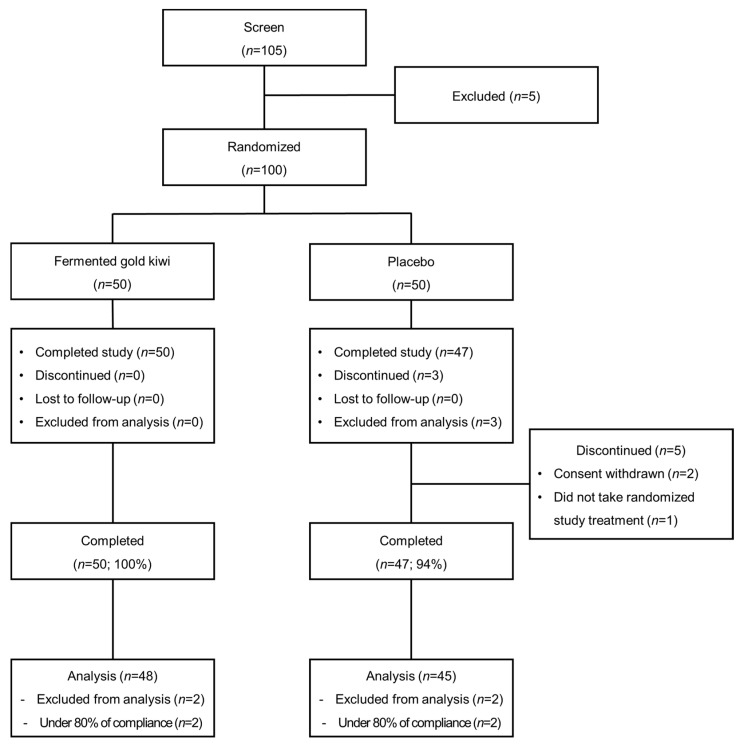
Randomized human clinical study participant disposition.

A total of 105 participants completed the written consent form and underwent testing, resulting in the identification of a final cohort of 100 individuals who met the eligibility criteria. A total of 47 participants in the Placebo group and 50 participants in the FGK group, accounting for more than 94% of total participants, successfully completed the study (Figure 9). A total of 47 participants in the Placebo group and 50 participants in the FGK group were included in the final analysis. Two participants from the FGK group and two participants from the Placebo group were excluded from the analysis.

The participants’ demographic details, including their sex, age, height, weight, and body mass index, are presented in Table 5.

### 3.8. GSRS Score

#### 3.8.1. Total Score for Lower Gastrointestinal Symptoms

**Figure 10:**
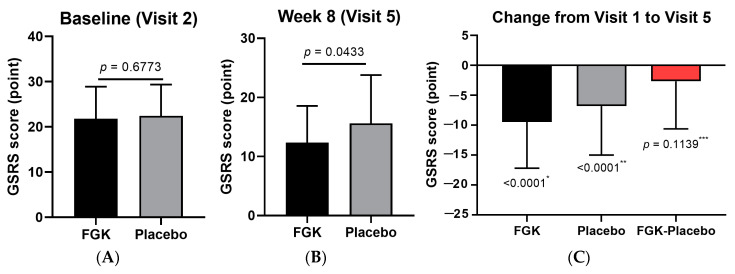
Changes in the GSRS lower gastrointestinal part before (baseline) and after (8 weeks) administration of FGK. (**A**) GSRS lower gastrointestinal part score at visit 2 (baseline); (**B**) GSRS lower gastrointestinal part score at visit 5 (8 weeks); (**C**) the change from visit 2 to visit 5. Data are expressed as the mean ± SD. (**A**) Comparisons between the groups using the *p*-values from two-sample *t*-tests. (**B**) Comparisons between the groups using Wilcoxon signed-rank tests. *^,^** Comparisons within the groups using paired *t*-tests. *** 95% two-sided confidence interval for differences in the least squares mean.

At baseline (visit 2), no significant differences were observed in the lower gastrointestinal symptom scores between the FGK and Placebo groups, as shown in Figure 10A. However, by week 8 (visit 5), the FGK group showed significantly lower scores (12.33 ± 6.23) compared to the Placebo group (15.60 ± 8.20), as shown in Figure 10B. The change from visit 2 to visit 5 was −9.46 ± 7.72 in the FGK group, compared to −6.80 ± 8.19 in the Placebo group (Figure 10C), indicating a more significant improvement in the FGK group.

#### 3.8.2. Constipation Symptoms

**Figure 11:**
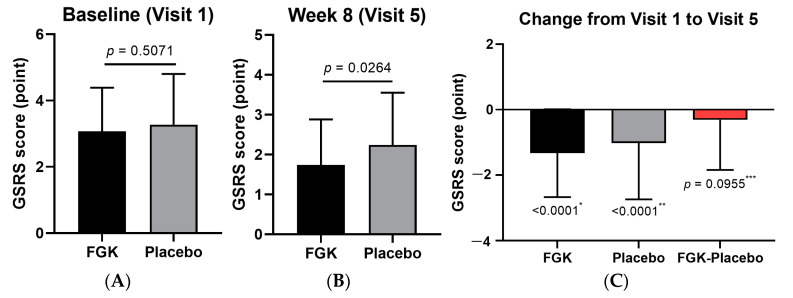
Changes in GSRS constipation symptoms before (baseline) and after (8 weeks) administration of FGK. (**A**) GSRS constipation symptom score at visit 2 (baseline); (**B**) GSRS constipation symptom score at visit 5 (8 weeks); (**C**) the change from visit 2 to visit 5. Data are expressed as the mean ± SD. (**A**) Comparisons between the groups using the *p*-values from two-sample *t*-tests. (**B**) Comparisons between the groups using Wilcoxon signed-rank tests. *^,^** Comparisons within the groups using paired *t*-tests. *** 95% two-sided confidence interval for differences in the least squares mean.

The constipation symptom scores were similar between the FGK group (3.07 ± 1.32) and the Placebo group (3.27 ± 1.53) at baseline (visit 2), as shown in Figure 11A. By week 8 (visit 5), the FGK group showed a greater reduction in constipation symptom scores (−1.33 ± 1.34) compared to the Placebo group (−1.02 ± 1.72), with final scores of 1.74 ± 1.14 and 2.24 ± 1.31, respectively (Figure 11B,C).

#### 3.8.3. Sensations of Not Completely Emptying the Bowels

**Figure 12:**
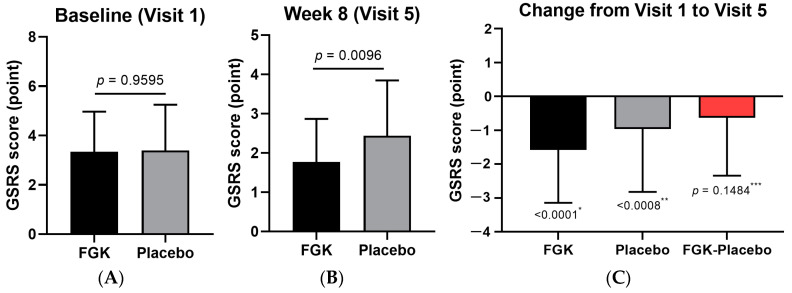
Changes in the GSRS sensation of not completely emptying the bowels before (baseline) and after (8 weeks) administration of FGK. (**A**) The sensation of not completely emptying the bowels score at visit 2 (baseline); (**B**) the sensation of not completely emptying the bowels score at visit 5 (8 weeks); (**C**) the change from visit 2 to visit 5. Data are expressed as the mean ± SD. (**A**) Comparisons between the groups using the *p*-values from two-sample *t*-tests. (**B**) Comparisons between the groups using Wilcoxon signed-rank tests. *^,^** Comparisons within the groups using paired *t*-tests. *** 95% two-sided confidence interval for differences in the least squares mean.

At baseline (visit 2), the sensation of incomplete bowel emptying was similar between the FGK (3.35 ± 1.62) and Placebo (3.40 ± 1.85) groups (Figure 12A). However, by week 8 (visit 5), the FGK group showed a significantly greater reduction in symptom scores (−1.58 ± 1.56) compared to the Placebo group (−0.96 ± 1.86), as shown in Figure 12B,C.

## 4. Discussion

The prokinetic effects of FGK (delayed gastric emptying, reduced gastrointestinal motility, gastric acid secretion, and constipation) were evident in four animal models and a human randomized clinical trial. FGK, when administered in a dose-dependent manner, stimulated gastric motility into cisplatin-induced smooth gastric emptying. The findings of this study are consistent with those of previous reports, indicating that the intraperitoneal administration of cisplatin delays gastric emptying [32,33]. Cisplatin contributes to delayed gastric emptying by releasing 5-HT3R in the gastrointestinal mucosa and inducing gastric relaxation [41]. The inhibitory effect of cisplatin on gastric emptying was significantly alleviated by pretreatment with FGK, which is believed to be attributable to the antioxidant properties of FGK. Atropine, as an anticholinergic agent, inhibits gastric motility, thereby reducing gastrointestinal motility [42]. Pretreatment with FGK was observed to alleviate the suppression of gastrointestinal motility induced by atropine, similar to its ability to mitigate the delayed gastric emptying caused by cisplatin (Figure 3A,B). These results suggest that pretreatment with FGK can enhance gastrointestinal peristalsis in both an animal constipation model and participants with related disorders.

The findings from this study suggest that FGK has significant gastroprotective properties. The reduction in total acidity, free acidity, pepsin activity, and total gastric acid volume, alongside the increase in gastric pH observed after FGK administration, indicate that FGK can effectively mitigate the damaging effects of the increased gastric acidity caused by pyloric ligation. These protective effects appear to be dose-dependent, further highlighting the potential therapeutic role of FGK in managing gastric acidity. This aligns with previous studies suggesting that plant-based compounds can exert gastroprotective effects by modulating gastric secretions and pH levels [29,43]. The ability of FGK to reduce gastric acidity and increase pH may be attributed to its bioactive components, which could play a role in inhibiting excessive gastric acid production and enhancing the mucosal defense mechanisms. Further research could focus on identifying the specific bioactive compounds responsible for these effects and exploring their potential applications in treating gastric disorders.

Maintaining balance in the gut microbiota is essential. Approximately 25–54% of the content of fecal matter is composed of bacteria, with different species of bacteria in the digestive system playing a crucial role in preserving intestinal equilibrium, aiding in the production of vitamins, supporting immune responses, and regulating hormonal functions [44,45]. Disruption of the equilibrium of intestinal microorganisms can lead to constipation or diarrhea, which may cause various complications [46]. Several studies have documented that loperamide-induced constipation disrupts the composition of gut microbiota, damages the intestinal barrier, and impairs secretory function [47,48]. A recent study has shown that certain combinations of probiotics relieve constipation more effectively than the consumption of a single probiotic strain [49]. In addition, a reduction in constipation symptoms has been reported in patients who consume three gold kiwis daily [50]. Consistent with the findings of this study, a fermented product derived from gold kiwis was formulated with various probiotics and was subjected to experimental testing in both animal models and human participants. The study demonstrated the restoration of intestinal microbiota balance by enhancing the levels of *Lactobacillus* and *Bifidobacterium* while reducing *Enterobacteriaceae* levels in animals with loperamide-induced constipation.

The primary objective of this study was to evaluate the potential of FGK, through various probiotics, for improving gastrointestinal function and alleviating functional gastrointestinal disorders, such as constipation. Our previous research demonstrated the protective effects of FGK in rats with induced acute gastritis and provided evidence from clinical trials regarding its benefits for gastrointestinal health [21,37]. FGK’s ability to mitigate the effects of loperamide-induced constipation supports its role in enhancing gastrointestinal peristalsis and normalizing bowel movements. This finding aligns with previous studies, which have shown that drug administration, such as loperamide, leads to reduced fecal pellet size, decreased food intake, and lowered fecal moisture content [47,51]. The observed delay in fecal transit and increase in fecal pellet number further support the reliability of the loperamide-induced constipation model for replicating spastic constipation symptoms. This model provided a strong basis for assessing the potential of FGK for alleviating constipation and improving gastrointestinal function. Previous studies have suggested that changes in the abundance of *Lactobacillus* species and *Bifidobacterium* species contribute to the alleviation of constipation symptoms [47,52]. The observed changes in gut microbiota, including increased *Lactobacillus* and reduced *Enterobacteriaceae* abundance, further suggest that FGK may restore intestinal barrier function and alleviate the symptoms of constipation. Previous research has emphasized the importance of gut microbiota in maintaining gut health, and FGK’s impact on bacterial composition underscores its therapeutic potential.

Enteroendocrine cells secrete 5-HT, which initiates peristaltic reflexes in the intestine [53], and it is one of the most important neurotransmitters mediating communication between the brain and the gut [54]. Serotonergic agents have been widely used to study the mechanisms underlying the pharmacological effects of treatments for constipation-related disorders [55]. Treatment with loperamide in rats blocks the 5-HT receptors, leading to a decrease in serum serotonin levels [9,56,57]. Among the 5-HT receptors, 5-HT3 and 5-HT4 play crucial roles in the mechanisms regulating gastrointestinal motility. Agonists of 5-HT3 have been shown to be effective in treating IBS associated with diarrhea, while 5-HT4 agonists have proven effective in treating IBS associated with constipation and chronic constipation [53]. In addition, 5-HT4 plays a role in gastrointestinal motility, mucosal secretion, and neuronal signaling [58,59]. It is found predominantly in the gastrointestinal tract, with approximately 95% located in the gut, where it is released from the enterochromaffin cells distributed throughout the intestinal mucosa [60]. In our study, rats treated with loperamide showed a decrease in the expression of 5-HT3 and 5-HT4 in rat colonic tissue (Figure 3A,B). This result is consistent with the observed reduction in serum serotonin levels (Figure 4A). In contrast, rats treated with FGK exhibited a dose-dependent increase in the expression of 5-HT3 and 5-HT4 in the colonic tissue, with a significant increase compared to the Lope group. Additionally, serum serotonin levels were significantly elevated in the FGK 250 group. These findings confirm the efficacy of FGK in alleviating constipation by modulating the levels of 5-HT3, 5-HT4, and serum serotonin.

Dopamine is a hormone that is closely associated with gut microbiota imbalance [61]. Its role in gastrointestinal motility involves binding to receptors and inhibiting the release of acetylcholine. As dopamine secretion increases, gastrointestinal motility reportedly decreases [9]. Similarly, other studies have found that a reduction in 5-HT induced by loperamide is closely related to a decrease in serum acetylcholine levels. Increasing the levels of 5-HT and acetylcholine should, therefore, be associated with improved gastrointestinal motility and a reduction in fecal accumulation in the intestines [62]. Consistent with previously reported studies, our research showed an increase in the expression of D2R in the colons of rats with loperamide-induced constipation (Figure 3C), and a corresponding increase in serum dopamine levels (Figure 4B). These increases have a significant impact on serum acetylcholine levels, which were markedly reduced in the Lope group (Figure 4C). FGK suppressed the loperamide-induced increase in D2R expression and decreased serum dopamine levels. In addition, serum acetylcholine levels were observed to increase. These findings suggest that FGK can alleviate constipation by influencing the relationship between gut microbiota and neurotransmitters.

Breakdown of the intestinal epithelial barrier due to loperamide administration leads to impaired intestinal function and subsequent constipation, which, in turn, increases the expression of pro-inflammatory cytokines [63]. The overexpression of pro-inflammatory cytokines, such as TNF-α, IL-1β, and IL-6, induces the disruption of connexins, such as claudin-1, between cells, leading to the collapse of intestinal structural integrity [64]. In this study, the expression of inflammatory cytokines in the intestine increased following loperamide treatment but decreased after FGK administration (Figure 5). This suggests that FGK can alleviate inflammatory responses in a loperamide-induced constipation model. However, a previous clinical trial found that FGK consumption did not significantly affect levels of TNF-α and IL-6 in the participants [37].

The clinical trial conducted in this study analyzed FGK’s effects on lower gastrointestinal health, focusing particularly on constipation symptoms. The Rome IV classification defines two main syndromes of functional dyspepsia: epigastric pain syndrome and postprandial distress syndrome [65]. However, it is known that some patients with functional dyspepsia also experience the symptoms of functional constipation [66]. This overlap may be attributed to the influence of gut–brain interactions on neurotransmitter levels, which affect both dyspepsia and constipation [53,60]. Evidence from healthy individuals indicates that the suppression of bowel movements can lead to delayed gastric emptying, highlighting a link between constipation and dyspepsia [67]. Additionally, irritable bowel syndrome, a common functional gastrointestinal disorder, is diagnosed using the clinical parameters outlined by the Rome IV criteria [68].

## 5. Conclusions

Our findings emphasize the alleviating effects of FGK on the symptoms of constipation through the promotion of bowel movements in both a constipation-induced rat model and participants suffering from symptoms of lower gastrointestinal disorders that are similar to constipation. In the animal model, FGK intake alleviated constipation by enhancing gastrointestinal motility, which can be attributed to the regulation of gut hormones and the suppression of inflammatory factors. Although the clinical trial did not show significant improvements in dyspepsia symptoms, these findings contribute to understanding the limitations of FGK in this population. These results suggest that FGK consumption can be an effective strategy for preventing and treating conditions related to gastrointestinal motility, such as delayed gastric emptying, reduced gastrointestinal motility, and constipation symptoms.

## Figures and Tables

**Table 1:** Lactic acid bacteria used for the fermentation process of FGK.

Bacterial Strains	Source	Origin
Lactococcus lactis VI-01 (KTCT 14351 BP)	Isolate	Kiwi peel
Lacticaseibacillus paracasei VI-02 (KTCT 14352 BP)	Isolate	Kiwi peel
Lacticaseibacillus casei VIGRA01 (KTCT 14756 BP)	Isolate	Cheese
Lactobacillus helveticus VICAM05 (KTCT 15949 BP)	Isolate	Cheese
Lactobacillus acidophilus VIFEC24 (KTCT 15950 BP)	Isolate	Infant feces

**Table 2:** Proximate composition of an FGK sample.

Composition	FGK
Calorie (Kcal/100 g)	41.14
Carbohydrate (%)	8.67
Crude protein (%)	1.48
Crude fat (%)	0.06
Moisture (%)	89.46
Crude ash (%)	0.33
α-Amylase (U/g)	121.5 ± 1.98
Lipase (U/g)	4.42 ± 0.01
Protease (U/g)	21.15 ± 0.07

**Table 3:** Primer sequences.

Gene Name	Sequence of PCR Primer (5′-3′)	
5-HT3R	F	ATTTTGTGGTGTGCATGGCT
	R	GCTCCCCTAGGCAGAGTATC
5-HT4R	F	GATGCTAATGTGAGTTCCAACGA
	R	CAGCAGGTTGCCCAAGATG
D2R	F	CACCACGGCCTACATAGCAA
	R	GGCGTGCCCATTCTTCTCT
TNF-α	F	TGATCCGAGATGTGGAACTG
	R	CGAGCAGGAGTAAGAAGAGG
IL-1β	F	TGACCCATGTGAGCTGAAAG
	R	GGGATTTTGTCGTTGCTTGT
IL-6	F	CCGGAGAGGAGACTTCACAG
	R	CCATAGTGCAGGAGCGTACAGT
GAPDH	F	TGACCTCAACTACATGGTCTACA
	R	CTTCCCATTCTCGGCCTTG
Total bacteria	F	GCAGGCCTAACACATGCAAGTC
	R	CTGCTGCCTCCCGTAGGAGT
*Lactobacillus* group	F	CGATGAGTGCTAGGTGTTGGA
	R	CAAGATGTCAAGACCTGGTAAG
*Bifidobacterium* group	F	CTCCTGGAAACGGGTGG
	R	GGTGTTCTTCCCGATATCTACA
Enterobacteriaceae	F	TGCCGTAACTTCGGGAGAAGGCA
	R	TCAAGGCTCAATGTTCAGTGTC

**Table 4:** Effects of FGK on body weight and feed intake in rats with loperamide-induced constipation.

Groups	Initial Body wt (g)	Final Body wt (g)	Gain Body wt (g/day)	Feed Intake (g/day)
Normal	207.9 ± 2.6 ^NS^	281.6 ± 5.5 ^NS^	6.14 ± 0.37 ^NS^	20.05 ± 0.17 ^ab^
Lope	209.1 ± 1.9	281.2 ± 3.2	6.02 ± 0.21	19.55 ± 0.02 ^b^
LAB	208.8 ± 3.1	284.7 ± 4.0	6.37 ± 0.20	20.59 ± 0.11 ^a^
FGK 50	208.3 ± 2.6	278.5 ± 3.7	5.85 ± 0.16	19.59 ± 0.04 ^b^
FGK 125	206.3 ± 2.5	272.6 ± 2.8	5.53 ± 0.14	20.21 ± 0.19 ^a^
FGK 250	206.6 ± 3.0	277.1 ± 4.1	5.87 ± 0.18	20.27 ± 0.10 ^a^

Results were represented as the mean ± SEM (n = 6). ^a,b^ Different letters indicate statistically significant differences in the same column (*p* < 0.05). ^NS^ No significant differences among the group. Normal, no-constipation rats; Lope, loperamide-induced constipation in rats; LAB, lactic acid bacteria; FGK, fermented gold kiwi.

**Table 5:** Demographic characteristics of the trial subjects.

	FGK (*n* = 50)	Placebo (*n* = 47)	*p*-Value
Sex (M/F)	12/36	7/38	0.2589 (C) ^1^
Age (year)	45.17 ± 10.53	40.67 ± 11.74	0.0545 (T) ^2^
Height (cm)	162.95 ± 8.52	162.48 ± 8.38	0.4307 (W) ^2^
Weight (kg)	64.36 ± 11.30	61.06 ± 13.63	0.0522 (W) ^2^
BMI (kg/m^2^)	24.11 ± 2.97	23.02 ± 4.06	0.0136 (W) ^2^

^1^ Compared between groups: *p*-value determined by chi-square test (C). ^2^ Compared between groups: *p*-value by a two-sample *t*-test (T) or Wilcoxon rank sum test (W).

## Data Availability

The data presented in this study are available on request from the corresponding author. The data are not publicly available due to ethical restrictions.
